# Prevalence of psychosocial work factors and stress and their associations with the physical and mental health of hospital physicians: A cross-sectional study in Lithuania

**DOI:** 10.3389/fpubh.2023.1123736

**Published:** 2023-02-13

**Authors:** Rasa Zutautiene, Gintare Kaliniene, Ruta Ustinaviciene, Ricardas Radisauskas

**Affiliations:** ^1^Department of Environmental and Occupational Medicine, Faculty of Public Health, Lithuanian University of Health Sciences, Kaunas, Lithuania; ^2^Health Research Institute, Faculty of Public Health, Lithuanian University of Health Sciences, Kaunas, Lithuania

**Keywords:** psychosocial risk, stress, mental health, physical health, physician

## Abstract

**Background:**

A negative psychosocial work environment causes stress to the physicians of healthcare institutions, which affect their physical and mental health. This study aimed to evaluate the prevalence of psychosocial work factors and stress and their associations with the physical and mental health of hospital physicians in the Kaunas region of Lithuania.

**Materials and methods:**

A cross-sectional study was performed. It was based on a questionnaire survey, which contained the Job Content Questionnaire (JCQ), three scales of Copenhagen Psychosocial Questionnaire (COPSOQ), and Medical Outcomes Study Short Form-36 (SF-36) health survey. The study was carried out in 2018. A total of 647 physicians completed the survey. Multivariate logistic regression models were performed by using the stepwise method. In the models potentially, confounding factors such as age and gender were controlled. In our study, the dependent variables were stress dimensions, and the independent variables were psychosocial work factors.

**Results:**

The analysis showed that a quarter of surveyed physicians were classified as having low job skill discretion and decision-making authority, and the support of supervisors was weak. Approximately one-third of the respondents had low decision latitude, low co-worker support, and high job demands, and felt insecure at work. Job insecurity and gender were found to be the strongest independent variables for general and cognitive stress. The support of the supervisor was found as a significant factor in the case of somatic stress. Better evaluation of mental health was related to job skill discretion and co-workers' and supervisors' support, but it did not affect physical health.

**Conclusion:**

The confirmed associations suggest that looking at work organization factors, reducing exposure to stress, and increasing perception of the psychosocial environment can be linked to better subjective health evaluation.

## 1. Introduction

According to the World Health Organization, one of the biggest current challenges is improving the health of workers and managing the psychosocial factors in the work environment of organizations (1). The European Agency for Safety and Health at Work estimates that 50–60% of all working days are lost due to stress at work and psychosocial risks (2).

According to WHO, the definition of health is a holistic approach to a worker's health. Attitudes toward work environment factors (from physical, chemical, ergonomic, and biological) have changed in recent years, and psychosocial work environment factors are increasingly being discussed (1). Psychosocial work factors include lack of time to do work, high requirements, low control at work, lack of support from co-workers and supervisors, and insecurity at work. They cause job strain, mental stress, and stress to employees, which result in physical and mental health problems. The term “physician wellbeing” refers to career opportunities, life satisfaction, a significant meaning in life, and a commitment both to direct patient care and clinical practice (3), but research on the occupational health of employees usually focuses on occupational risk factors, and more rarely on the creation of the positive working environment. For working people to be physically and mentally healthy and to feel satisfied with their work, it is important to take care not only of occupational safety and health but also of wellbeing at work. The quality of work–life is a major issue for the sustainability of career tracks among physicians (4).

Job strain is a stressful situation that occurs as a result of physical and psychological pressures that the employee feels in the process of fulfilling what is expected by the workplace. The physical expressions caused by job strain are digestive tract and sleep disorders. Health workers are considered to be at high risk. (5). The prevalence of job strain among physicians of different specialties is not widely studied. There are some scientific publications examining job strain and burnout among physicians of different specialties. For example, among French emergency physicians, the job strain was 17–32% (4), the job strain among physicians in Germany of the specialty of psychiatry was 58.5% (6), and the job strain in primary care was 30% of GPs in the United Kingdom (7). In Turkey, ~24.5% of family physicians feel burnout (8). A study of Lithuanian anesthesiologists and intensive care specialists showed high rates of burnout, closely related to high workload and low salary (9, 10), and a study of family physicians showed a high level of bullying in the workplace (11).

Scientific publications emphasize that negative psychosocial work factors have a significant impact on the physical and mental health of workers. Working conditions in hospitals are often characterized as stressful and detrimental to health (12, 13). They cause the following physical and mental health problems: tension (14), exhaustion, anxiety (15), depression (16), suicidal thoughts (1), cardiovascular diseases (17), and longer post-discharge recovery times (17–19).

The improvement of working conditions should be systematically researched. There is still a lack of research in Lithuania on the psychosocial work environment of doctors, stress, and its impact on mental and physical health. Therefore, we focus on physicians of various sectors in six hospitals in the Kaunas region of Lithuania. This investigation aims to reveal hospital physicians' situations.

This study aimed to evaluate the prevalence of psychosocial work factors and stress and their associations with the physical and mental health of hospital physicians in the Kaunas region, Lithuania.

## 2. Materials and methods

### 2.1. Study design and participants

This cross-sectional study was carried out from September to December 2018 in six hospitals in the Kaunas region of Lithuania. Participation in the research of physicians was voluntary and anonymous. The study population (*N* = 2,353) included all physicians working in hospitals. The sample size calculation was based on the frequency with a 5% probability of error and 95% reliability, and 0.5 relative frequency (20), and this resulted in 330 participants needed to complete the study. A total of 830 questionnaires were distributed among physicians, and the final sample size was 647 respondents, (response rate −81.3%). In our study, purposive sampling was used. The six largest hospitals in the Kaunas region were selected. In these hospitals, questionnaires were distributed to every third department. A pilot study was conducted. A total of 109 respondents filled out the questionnaire correctly. Correctly completed questionnaires were included in the database of the main study. The dispersion of responses obtained after the pilot study met the statistical criteria.

All study variables were assessed *via* standardized questionnaires. The study protocol was approved by the Kaunas Regional Ethics Committee for Biomedical Research (Protocol No. BE-2-41).

### 2.2. Questionnaires

#### 2.2.1. Assessment of sociodemographic characteristics

A four-part questionnaire was used in this study. The first part of the questionnaire revealed the sociodemographic characteristics of the respondents (gender, age, length of employment, workload, specialties, and night work).

#### 2.2.2. Assessment of psychosocial factors

Information about work environment characteristics was obtained from the second part of the questionnaire: The Job Content Questionnaire (JCQ) (21). This instrument had been designed in 1979 by R. Karasek to measure work environment characteristics, is a well-established and widely used self-report instrument based on the demand-control-support model. The JCQ comprises five scales: job demand (five items), job control (nine items—the sum of two subscales: skill discretion measured by six items and decision authority measured by three items), supervisor support (four items), co-worker support (four items), and job insecurity (three items). Items are scored using a Likert scale in which 1 indicates that the respondent strongly disagrees and 4 indicates that he or she strongly agrees, except for the job insecurity scale's questions with different possible answers that are rated on a five-point scale. Work environment characteristics based on the demand-control-support model and its associations with burnout we presented in our previous publication (22), and this study is focused on associations between psychosocial work factors, stress, and physical and mental health among physicians.

#### 2.2.3. Assessment of health outcomes

The third part of the questionnaire was a standardized Medical Outcomes Study Short Form-36 health survey (23, 24). It is a 36-item general health questionnaire designed to provide physical and mental health summary scores based on eight subscales: physical functioning, role functioning, bodily pain, general health assessment, vitality, social functioning, activity restriction on emotional disorders, and emotional state. In these questions, we sought to assess physicians' subjective health status over the past 4 weeks. In this study, we used only derived values—physical (PSC) and mental (MSC) health. PSC health consists of four subscales (physical functioning, role functioning, bodily pain, and general health assessment) summary scores. MSC health consists of four subscales (vitality, social functioning, activity restriction on emotional disorders, and emotional state) summary scores. According to the methodology of the questionnaire, the variants of the answers were transferred to the scoring system from 0 to 100. A higher score indicates a better quality of life. The evaluation of each scale corresponded to the calculated average of the scores on the score scale. It is divided according to the limits of terciles—high (100–66.7 points), medium (66.7–33.3 points), or low (33.3–0 points) levels of the observed phenomenon.

#### 2.2.4. Assessment of stress in the work environment

The Copenhagen Psychosocial Questionnaire (COPSOQ) was used to assess physicians' perceived stress in the work environment. The COPSOQ was developed in Denmark in 2000 as a screening instrument for recording psychological stress and stress at work and revised in 2005. In this study, we used three scales of stress expression: general stress (eight questions), somatic stress (seven questions), and cognitive stress (four questions) (25). We replaced the answers to the questions with numbers from 0 to 100. The evaluation of each scale corresponded to the calculated average of the scale scores. These estimates were then divided into three parts according to the limits of the terciles—high (100–66.7 points), medium (66.7–33.3 points), or low (33.3–0 points) levels of the observed phenomenon.

### 2.3. Statistical analysis

All the statistical analyses were performed using the IBM SPSS 25.0 software package (IBM Inc., Armonk, New York, NY, USA). Descriptive data were expressed as a percentage, mean, median, standard deviation (SD), 95% confidence intervals (CIs), and min/max.

To find out associations between psychosocial work environment factors (such as job demand, job control, co-worker and supervisor support, and job insecurity) and stress dimensions, a multivariate logistic regression model using the stepwise method was applied. In the models, potentially confounding factors such as age and gender were controlled. Controlling the dependent variables were stress dimensions and the independent variables were job demand, job control, supervisor support, co-worker support, and job insecurity.

Finally, to determine the association between psychosocial work factors, stress, and physical and mental health, three models of the multiple stepwise regression analyses were performed for each self-rated health subscale (physical and mental). The bad health of each binary outcome variable (PSC and MSC) was chosen as a reference group in the model of logistic regression. Potentially confounding factors such as age and gender were controlled. In all three models, physical and mental health were dependent variables and the independent variables were in model I—psychosocial work environment factors, in model II—stress subscales, and in model III—both psychosocial factors and stress dimensions, respectively.

The results are presented as regression coefficients (B), odds ratios (ORs), 95% confidence intervals (CIs), and *P*-value. The accuracy and feasibility of multivariate logistic regression models were evaluated using the classification table and the Nagelkerke *R*^2^ test (*R*^2^_*N*_).

A significance level of 0.05 was selected. Differences and relationships were considered to be significant if *p* < 0.05.

## 3. Results

The main sociodemographic and occupation-related characteristics of participants are shown in Table 1. The biggest part (65.7%) of the study participants was female. The mean age of the study sample was 39.7 (SD = 13.58) years and (57.7%) ranged in age from 24 years to 40 years. Participants had been working at their current job for an average of 14 years (SD = 13.19). Approximately one-third (35.5%) of respondents were attributed to the work experience group for 4 years. The main proportion (51.3%) consisted of respondents living in partnership (married), more than a third (37.1%) were single, and others were divorced and widowed. Slightly more than half of the respondents were therapeutic profile specialists, 25.2% were surgical, and 22% attributed to other specialties.

**Table 1:** The main sociodemographic and occupation-related characteristics of the study population.

**Characteristic**	***N* (%)**
**Gender**	
Men	222 (34.3)
Woman	425 (65.7)
**Age group (years)**	
<=40	373 (57.7)
41–50	104 (16.1)
>50	170 (26.2)
**Length of employment (year)**	
<=10	343 (53.0)
11–30	212 (32.8)
>30	92 (14.2)
**Workload**	
<=1	171 (26.4)
>1	476 (73.6)
**Specialties**	
Surgical	163 (25.2)
Therapeutic	340 (52.6)
Other (not specified)	144 (22.2)
**Night work**	
Yes	455 (70.3)
No	192 (29.7)

Descriptive statistics of all study variables are presented in Table 2. The analysis showed that the psychosocial work environment was quite negative in investigated physicians population—approximately a quarter of them were classified as having low job skill discretion and decision-making authority as well as the support of the supervisor was weak. Approximately one-third of respondents had low decision latitude, low co-worker support, high job demands, and felt insecure at work. The analysis of stress evaluation showed that the highest mean of scale scores was in the case of general stress, and the frequencies of low- and high-stress levels (cut point—median) showed that approximately half of the respondents were classified as perceived high-stress levels in all three stress evaluation scales. The scores of PSC and MSC health were categorized into two groups: high/intermediate (good/intermediate health) and low (poor health). A majority of respondents were classified as good and intermediate health in both dimensions of PSC and MSC health-−66.8 and 67.1%, respectively. Further analysis showed that the frequency of poor PSC was significantly higher in women compared with men (36.2% in women vs. 27.5% in men, *p* < 0.05).

**Table 2:** Descriptive statistics of psychosocial work environment and perceived health characteristics.

**Characteristic**	**Mean (SD)**	**n (%)**	**Median**	**Min–max**
**Job skill discretion**	36.18 (5.13)		36.00	20–48
Low		*149 (23.0)*		
Moderate		*212(32.8)*		
High		*286 (44.2)*		
**Job decision-making authority**	34.75 (6.54)		36.00	12–48
Low		*144 (22,2)*		
Moderate		*115 (17.8)*		
High		*388 (60.0)*		
**Job demand**	33.00 (4.80)		33.00	18–48
Low		*113 (17.5)*		
Moderate		*300 (46.4)*		
High		*234 (36.1)*		
**Job decision latitude**	70.00 (10.30)		68.00	32–96
Low		*187 (28.9)*		
Moderate		*202 (31.2)*		
High		*258 (39.9)*		
**Co-worker support**	12.00 (1.62)		12.00	4–17
Low		*187 (28.9)*		
Moderate		*306 (47.3)*		
High		*154 (23.8)*		
**Supervisor support**	11.53 (2.37)		12.00	4–18
Low		*168 (26.0)*		
Moderate		*54 (8.3)*		
High		*425 (65.7)*		
**Job insecurity**	5.38 (1.63)		5.00	3–12
Low		*195 (30.1)*		
Moderate		*217(33.5)*		
High		*235 (36.4)*		
**General stress**	34.37 (21.19)		34.37	0–96.9
Low		*318 (49.1)*		
High		*329 (50.9)*		
**Cognitive stress**	20.57 (15.25)		25.00	0–87.5
Low		*356 (55.0)*		
High		*291 (45.0)*		
**Somatic stress**	27.22 (19.33)		17.85	0–75.0
Low		*299 (46.2)*		
High		*348 (53.8)*		
**Physical health**	63.44 (10.39)		63.75	26.53–92.5
Good		*432 (66.8)*		
Poor		*215 (33.2)*		
**Mental health**	55.87 (10.32)		55.75	13.78–82.5
Good		*434 (67.1)*		
Poor		*213 (32.9)*		

At first, the association between psychosocial work environment factors and stress dimensions was assessed. As shown in Table 3, job decision-making authority and supervisor support significantly reduced general stress probability while job demand and job insecurity increased, the strongest variables for general stress were the controlled variables such as gender (women were more prone to have general stress) (*B* = 0.615) and job insecurity (*B* = 0.23). Job skill discretion, job demand, job insecurity, and gender were found as significant variables for cognitive stress in the studied population. As in the case of general stress and cognitive stress, the significant factors were gender and job insecurity (B, respectively, was 0.672 and 0.176). The support of the supervisor was found as the strongest factor in the case of somatic stress, and it was observed as a stress-buffering effect of this variable (*B* = −0.243). In addition, other factors such as job decision-making authority, job demand, job decision latitude, and job insecurity were significant variables of somatic stress. Gender was not significant for somatic stress, as seen in the model.

**Table 3:** Relationships between psychosocial work factors and stress expressions (multivariate comparison).

		**B**	**OR**	**95% CI**	**p**
General stress^*^	Job decision-making authority	−0.044	0.957	0.932–0.982	<0.001
	Job demand	0.054	1.051	1.017–1.096	0.004
	Supervisor support	−0.158	0.85	0.791–0.922	<0.001
	Job insecurity	0.233	3.095	1.851-5.175	<0.001
	Gender	0.615	1.849	1.300-2.630	<0.001
	^*^Classificationtable66.1%; *NagelkerkeR*^2^ = *0.17*				
Cognitive stress^*^	Job skill discretion	−0.035	0.96	0.935–0.998	0.03
	Job demand	0.054	1.056	1.018–1.095	<0.001
	Job insecurity	0.176	1.192	1.069–1.330	<0.001
	Gender	0.672	1.959	1.395-2.750	<0.001
	^*^Classification table 68.0%; Nagelkerke R^2^ = 0.20				
Somatic stress^*^	Job decision-making authority	−0.103	0.902	0.845–0.964	0.002
	Job demand	0.084	1.087	1.046–1.131	<0.001
	Job decision latitude	0.043	1.043	1.000–1.088	0.048
	Supervisor support	−0.243	0.784	0.721–0.853	<0.001
	Job insecurity	0.155	1.167	1.040–1.310	0.008
	^*^Classification table 61.1%; Nagelkerke R^2^ = 0.09				

* Logistic regression model accuracy and feasibility.

Second, three models of the multiple stepwise regression analyses were performed for each self-rated health subscale (PSC and MSC) (Table 4). Model I revealed that only job decision latitude was significantly associated with PSC—each one-unit increase of this variable reduced the probability of bad PSC by an average of 2.8%. Meanwhile, the model I for MSC shows that three psychosocial factors such as job skill discretion, co-worker support, and supervisor support were found to have a buffering effect and reduced bad MSC odds by an average of 3.5, 14.9, and 11.4%, respectively. In model II, somatic stress and general stress increased bad PSC probability by an average of 2.5 and 1%, respectively. The general stress was found as a significant factor for MSC and increased bad MSC probability by an average of 3.1%. The somatic stress was the strongest independent variable for PSC in the final model III, even though the beta coefficient was very low (*B* = 0.023)—somatic stress increased bad PSC probability by an average of 2.3%, and all other factors, which were found significant in the previous models remained significant as well as job decision latitude reduced the probability of bad PSC by an average of 2.3%, but general stress increased bad PSC by an average of 1.1%. The strongest variable for MSC was co-workers' support (*B* = −0.153), it reduced bad mental health probability by an average of 14.2%. Furthermore, job skill discretion and general stress remained significant in the final model. Job skill discretion was found as the factor, which reduced the probability of bad mental health by an average of 5%, and contrary to this, general stress was found as a bad MSC increasing factor (OR = 1.029 95% CI = 1.021–1.038).

**Table 4:** Association between psychosocial work factors, stress, and physical and mental health (multiple stepwise regression analyses).

			**B**	**OR**	**95% CI**	***P***
**PSC** ^^*^^	Model I	Age	0.017	1.017	1.005–1.030	0.007
		Gender	0.431	1.540	1.074–2.207	0.019
		Job decision latitude	−0.029	0.972	0.955–0.988	0.001
	^^**^^Classification table 66.8%; Nagelkerke R^2^ = 0.04					
	Model II	Somatic stress	0.025	1.025	1.013–1.037	<0.001
		General stress	0.010	1.010	1.001–1.019	0.026
	^^**^^Classification table 67.1%; Nagelkerke R^2^ = 0.07					
	Model III	Age	0.015	1.015	1.002–1.028	0.022
		Job decision latitude	−0.027	0.974	0.957–0.991	0.003
		Somatic stress	0.023	1.023	1.011–1.036	<0.001
		General stress	0.011	1.011	1.002–1.020	0.016
	^^**^^Classification table 67.7%; Nagelkerke R^2^ = 0.1					
**MSC** ^^*^^	Model I	Age	−0.13	0.987	0.974–1.000	0.042
		Job skill discretion	−0.036	0.965	0.933–0.998	0.037
		Co-worker support	−0.161	0.851	0.754–0.961	0.009
		Supervisor support	−0.121	0.886	0.819–0.959	0.003
	^^**^^Classification table 66.8%; Nagelkerke R^2^ = 0.08					
	Model II	General stress	0.030	1.031	1.022–1.039	<0.001
	^^**^^Classification table 68.3%; Nagelkerke R^2^ = 0.1					
	Model III	Co-worker support	−0.153	0.858	0.767–0.961	0.008
		Job skill discretion	−0.051	0.950	0.917–0.985	0.005
		General stress	0.029	1.029	1.021–1.038	<0.001
	^^**^^Classification table 70.5%; Nagelkerke R^2^ = 0.15					

* A reference group bad health.

** Logistic regression model accuracy and feasibility.

## 4. Discussion

Physician burnout is an under-recognized and under-reported problem. In the scientific literature, the health of nurses is analyzed more often, and the links between the psychosocial work environment of physicians and physical and mental health are analyzed less often. The literature shows that patient care workers had significantly higher demands in comparison with other hospital staff. At the same time, a comparison of physicians and nurses shows different work environment elements and different stress levels. The differences are caused by the unequal level of responsibility for the patient's health, the role in patient treatment, the variety of work tasks, and patients with multiple pathologies. Stress is a constant element in the daily life of physicians. So, we used an instrument, which is measuring a wide range of psychosocial work environment aspects and also distinguished three types of stress. The main research question was to explore the prevalence of psychosocial risk factors at work and their relationship with stress levels. Second, we wanted to understand whether stress reactions are related to self-rated physical and mental health among hospital physicians. Our results suggest that measured psychosocial work environment factors are associated with hospital physicians perceived work-related psychosocial stress and strain. According to our results, we can see that several essential factors determine stress reactions. One of them is a personal factor—the female gender. In our study, women experience more stress than men. In the scientific literature, we can find contradictory opinions, which can be caused by a number of cultural factors. In the studies, Sharma et al. (26) and Faraji et al. (27) reported that men were likely to suffer from occupational stress could be due to the fact that men likely to assume more social or family responsibilities in traditional Asian cultures, and the managers of hospitals tend to assign more work to male medical workers. The principles of gender equality prevail in our country, and the differences could be caused by additional women's duties at home (28, 29) and the higher sensitivity of women to the factors of the work environment. A study by Jagsi et al. demonstrated that women were also more likely to experience gender bias and sexual harassment in their careers (28, 30). It may be an additional factor leading to higher stress levels in women.

However, we were trying to find the answer to the question: Which factor is most associated with overall stress and/or overall dissatisfaction with the work environment? According to our evaluation, job insecurity is one of the essential factors in the work environment determining the development of stress. This supports the hypothesis that an increased workload and negative factors in the psychosocial environment can lead to more job-related stress and strain, which may manifest in changes in the physical and mental health of doctors (17, 19, 29).

However, it is still difficult to answer the main question: Which factor is fundamental in terms of overall stress and dissatisfaction in the psychosocial work environment? At the same time, we can see that factors work in the opposite direction. These are elements of the work environment that have a positive effect and reduce the likelihood of stress. In our study, there are two factors: supervisor support and job decision-making authority. We can state that the combination of favorable and unfavorable psychosocial factors for health is different for each individual. However, by summarizing them, it is possible to refine the organization's problems and management features and finds optimal solutions for improving the working environment.

The set of positive and negative stressors is different in each research study or healthcare facility evaluated (3, 4, 17, 18). However, it is necessary to identify, as it creates the conditions for successful interventions in the work environment for better occupational health of the staff. An unfavorable psychosocial climate leads to a number of other problems, which, at the same time, reduce the prestige of the institution and the quality of work (31, 32). A favorable psychosocial environment leads to better feedback from employees and patient satisfaction with the institution's health care services.

We found some differences in evaluating stress, psychosocial work environment, and physical and mental health dimensions. Age has a positive effect on subjective mental health but negative associations with physical health. The factor related to better subjective health evaluation (both physical and mental) was job decision latitude. Better evaluation of mental health was related to job skill discretion and co-workers' and supervisors' support, but it had no effect on physical health. General stress had a negative effect on subjective health in even four evaluation models. This is in line with data from other studies performed in educated, industrialized, rich, and democratic societies (23, 33), but the same findings present studies performed in developing countries (31).

In summary, we found that the psychosocial working environment was associated with subjective health. Looking at work organization factors, reducing exposure to stress, and increasing perception of the psychosocial environment can lead to better subjective health evaluation. However, our results can reveal the main factors in the development of stress in a specific physician work environment. The administration of healthcare facilities can create intervention systems in workplaces. This would be a tailored solid basis for the development of workplace interventions built on strengths in the psychosocial environment and overcoming negative stressors at work.

Our study is contributing to the literature on physician psychosocial work environment and stress. However, there are some limitations. First, it is regression results from a cross-sectional study and does not suggest causal relationships. It would be preferable to conduct a follow-up study to reveal the causal relationship between the psychosocial work environment, stress, and self-rated health. Second, the health of workers was assessed using the SF-36 subjective health questionnaire. However, this questionnaire is widely used and well-regarded by many researchers (24, 33, 34). Third, the representativeness of the study sample needs to be improved. This study cannot be applied to the whole of Lithuania because our study area is limited to Kaunas county. In the future, we plan to expand the scope of research by conducting a representative assessment of the country. The fourth limitation is related to the personal life of investigated persons. It was not possible to rule out the impact of other stressogenic factors on the respondents due to their lifestyle in the home environment and personal life. As we found in our study, the coefficients of determination in the presented models were quite small.

## 5. Conclusion

For Lithuanian physicians, general and somatic stress were negatively related to job decision-making authorities and supervisor support and cognitive stress to job skill discretion. The general and somatic stress were directly related to job demand, job insecurity, and female gender for general stress and job decision latitude for somatic stress. The aging, female gender, and general and somatic stresses increased the probability of PSC and the age (being younger), lowering job skill discretion, co-workers and supervisors support, and rising general stress increased MSC. By assessing the psychosocial factors of the work environment of physicians, they can be adjusted, which in the future can lead to a better health status for doctors and indirectly lead to a higher quality of health services for patients.

## Data availability statement

The raw data supporting the conclusions of this article will be made available by the authors, without undue reservation.

## Author contributions

RZ was responsible for methodology preparation, conduction of investigation, data analysis and validation, original draft preparation and supervision, and literature analysis. GK was responsible for methodology preparation, data analysis and validation, and original draft preparation and supervision. RU was responsible for the conceptualization of the manuscript, data analysis and validation, and reviewing and editing of the manuscript. RR was responsible for the conceptualization of the manuscript, data analysis and validation, reviewing and editing of the manuscript, and administration. All authors contributed to the article and approved the submitted version.
